# The future of quantum technologies for brain imaging

**DOI:** 10.1371/journal.pbio.3002824

**Published:** 2024-10-28

**Authors:** Daniele Faccio

**Affiliations:** School of Physics & Astronomy, Advanced Research Centre, University of Glasgow, Glasgow, United Kingdom

## Abstract

The neurosciences have pioneered the use of quantum technologies for sensing and imaging the brain. This Perspective discusses next-generation technologies that promise low-cost, wearable imaging devices with high spatial and temporal resolution.

## Teaser

The neurosciences have pioneered the use of quantum technologies for sensing and imaging the brain. Next-generation technologies promise routes towards low-cost, wearable imaging devices with high spatial and temporal resolution.

Much of the progress in our understanding of the brain and our ability to assess brain health relies heavily on magnetic resonance imaging (MRI) and quantum technologies that rely on quantum properties of matter or radiation such, e.g., nuclear spin, entanglement or detection of single quanta. These technologies are undergoing a renaissance in funding and research, which has led to some very exciting developments in recent years, for example, reconstructing images, videos [1,2], and the semantics of entire sentences [3] from brain activity and fMRI data alone. The goal of this perspective is to provide a brief overview of some quantum technologies that may have an impact, for example, on the wearability, precision, and cost of these brain reading approaches.

Magnetic resonance imaging (MRI) is a noninvasive approach that relies on a quantum effect whereby charged particles can have a spin and thus interact with an external magnetic field that provides information about the conformation of the spins and of the matter that we are probing. One form of MRI known as functional MRI (fMRI)—which measures brain activity by detecting small changes of blood flow in the brain—can now achieve sub-millimetre resolution, albeit typically with a 5 to 10 second time lag that results from the fact that it measures a blood oxygenation level dependent (BOLD) signal.

Another noninvasive technology known as magnetoencelphagraphy (MEG) instead measures the magnetic fields generated as a result of the time-varying voltage signals at the cellular level. The quantum technology utilised here lies not in the physics of the signal itself but rather in the detection, which is typically achieved using the extreme sensitivity of superconducting quantum interference devices (SQUIDs). MEG has lower spatial resolution compared to fMRI of order 3 mm but without the BOLD time-lag.

It is clear that using fMRI and MEG to image the brain at ever-finer detail is enabling a deeper understanding of how the brain operates. However, fMRI has relatively high costs that severely limit accessibility and the range of experiments is constrained by the fact that lying down in an MRI scanner is not a “natural” environment in which animals or humans operate. MEG based on SQUIDs is moderately more wearable but still expensive, mostly as a result of the experiments needing to be housed in special rooms that shield the instruments from the Earth’s magnetic field. Progress in this area has been made with the introduction of optically pumped magnetometers (OPMs) [4,5]. This too is a quantum technology lasers place a cloud of into a specific transition that is sensitive to external magnetic fields. It is possible to bring OPMs much closer to the head than traditional magnetometers (approximately 5 mm versus approximately 2 to 4 cm) and therefore reduce the amount of shielding required, bringing costs down significantly. OPMs are still a relatively new technology but studies indicate it may be possible to refine the technology so that it will not require any shielding at all [6] and/or possibly enable MEG to match the spatial resolution of fMRI. Further on the horizon, nitrogen vacancy (NV) centres in diamond crystals are yet another relatively modern quantum sensing technology that has shown extreme sensitivity to magnetic fields [7].

Beyond the use of light to read out the information in quantum sensors, we can also use light itself to directly measure brain activity via functional near-infrared spectroscopy (fNIRS). First introduced in the 1980’s [8], this approach relies on injecting light into the head, often through a fibre that is placed in contact with the scalp and connected with another fibre placed at a certain distance (a few millimetres to a several centimetres) away from the input fibre. fNIRS measures a signal that is biologically equivalent to the BOLD signal measured by fMRI yet with spatial resolution of approximately 1 cm and temporal resolution similar to fMRI (due to the BOLD signal delay of fMRI). It has the distinct advantage of being fully wearable and portable and experiments “in the wild” are already underway, for example, so-called hyperscanning experiments that measure the brain activity of multiple people interacting with each other [9], or real-time estimation of brain activity in surgeons during simulation training exercises [10].

One should not necessarily see fMRI, MEG, and fNIRs as competing with each other. Each technology will have its own space and will address very different imaging challenges. Indeed, we currently do not have a single technology that combines the best possible spatial (millimetre or sub-millimetre scale) and temporal (10 to 100 ms) resolution within a wearable, portable, and low-cost device. Looking forward, it seems possible that OPM or related magnetic-sensing approaches may achieve this highly ambitious goal because, unlike the other methods currently available, there is nothing in the physics that would prohibit this. The outstanding challenge here is to extract the brain signal from the external magnetic field sources, including the Earth or other sources that can be found in any modern lived environment. A very interesting area that is also worth following closely is the fusion of these quantum technologies with AI processing of the data as there are possible routes towards wearable devices with sufficient precision for brain reading and brain diagnostics, for example, by bringing together data from various imaging approaches.

**Fig 1:**
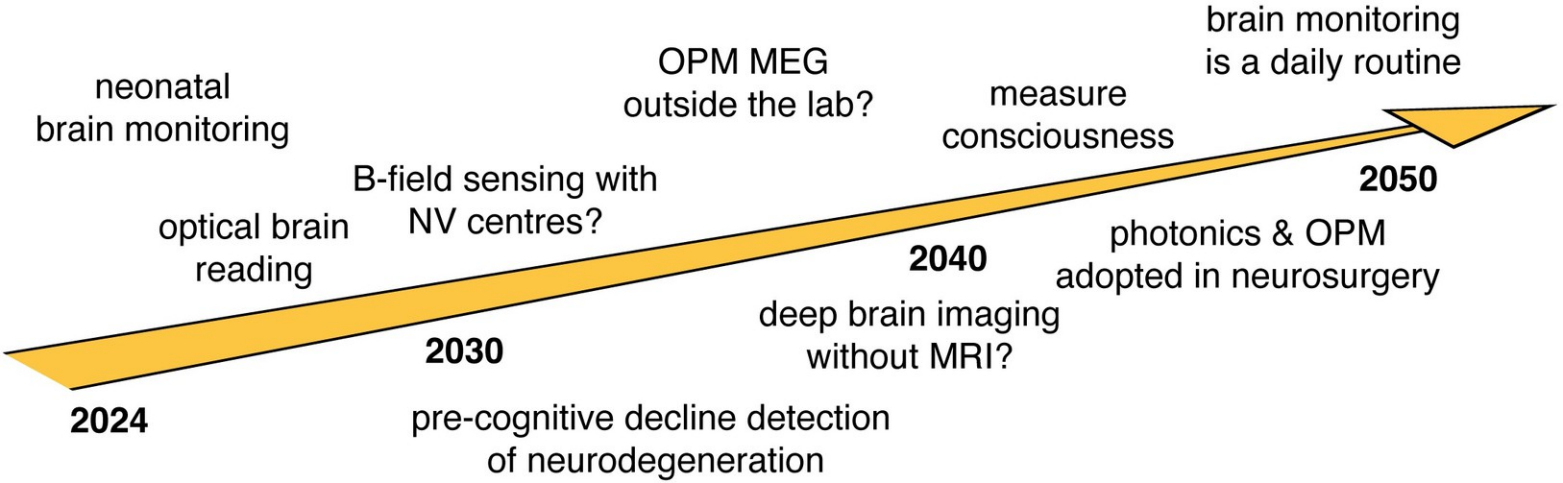
A speculative timeline of developments and applications of noninvasive neurotechnologies. This timeline and the list of technologies or applications are not meant to be complete but provide an outline of just some of the success stories that we hope to see over the next 10 to 20 years.

This brief overview of the main quantum technologies for brain imaging provides some insight into possible future directions for neuroscience. **Fig 1** gives a very partial overview of a possible timeline for the development of technologies and applications of noninvasive neurotechnology over the next 10 to 20 years. I believe fully wearable, low-cost devices are of particular value: what may we learn about the brain if we had imaging technologies that achieve similar functionality as fMRI, even with lower resolution, on individuals as they go about their usual lives?

At the lowest level, there is opportunity for monitoring brain health on a day-to-day basis, in much the same way that we currently use simple wearables that provide relatively precise health markers based on heart statistics. Simple electroencephalography wearables for the consumer market are currently available but often struggle with signal-to-noise ratio, something that can, in principle, be resolved by using fNIRS or one of the other technologies mentioned above. Interesting questions then revolve around identifying the exact brain metrics that correlate to cognitive performance, brain fatigue, overall brain health, and other measures of interest. Beyond this, regular brain monitoring providing detailed (spatial mapping with high-temporal resolution) brain data with a much lower barrier to access for both clinicians and patients may also soon become a reality. A question currently driving some research teams is whether it is possible to transfer some of the brain reading capability of fMRI to wearable fNIRS-based devices, an open challenge that, if solved, could have very profound consequences for the neurosciences and society.
